# Response to major peanut and peach allergens in a population of children allergic to peanut

**DOI:** 10.1186/2045-7022-5-S3-P128

**Published:** 2015-03-30

**Authors:** Alexandra Garcia-Blanca, Ana Aranda, Natalia Blanca-Lopez, Diana Perez, Maria Luisa Somoza, Francisca Gomez, Cristobalina Mayorga, Maria Jose Torres, Araceli Diaz Perales, Miguel Blanca, Gabriela Canto, Gador Bogas

**Affiliations:** 1 Allergy Service, Infanta Leonor Hospital, Madrid, Spain; 2 Research Laboratory, IBIMA, Regional University Hospital of Malaga, UMA, Malaga, Spain; 3 Allergy Service, Carlos Haya Hospital, Malaga, Spain; 4 Research Laboratory, Carlos Haya Hospital-FIMABIS, Malaga, Spain; 5 Centre for Plant Biotechnology and Genomic, Polytechnical University of Madrid, Madrid, Spain

## Introduction

Specific IgE to food allergens may vary according to geographic areas due to differences in dietary habits, environmental pollen exposure and other factors. Studies of the different patterns of sensitization, the relation with others allergens, clinical entities and variation according to age are needed. We studied sensitization to Arah2, Arah9 and Prup3 in a population of children and adolescents from 1 to 20 years, who were allergic to peanut and the relationship with peach allergy.

## Methods

Patients allergic to peanut were chosen from a large number of patients with allergy to plant-foods. They were classified in: A) those allergic to peanut with tolerance to peach and B) those allergic to peach and peanut. The IgE response was measured by ImmunoCAP to Arah2, Arah9 and Prup3 and the relationship with the different clinical entities as well as the variation according to age was analyzed.

## Results

From a total of 348 subjects evaluated, 28% were allergic to peanut. The median age was 10.48 years and 69% had sensitization to pollens, with *Phleum*, *Olea*, *Platanus* and *Artemisia* the most relevant. Urticaria appeared in 47%, followed by anaphylaxis (27%) and oral allergy syndrome (23%). A positive response to Arah2 and/or Arah9 appeared in the 59% of cases. The 54% reported symptoms with peanut and tolerance to peach (Group A) and 46% to peanut and peach (Group B). We observed significant differences in sex (p=0.02), age (p=0.01) and the sensitization to *Artemisia* (p=0.03). In both groups IgE response to the allergens was found, with predominance of Arah2 in group A and Arah9 (p=0.001) and Prup3 (p=0.008) in group B. In the different age groups observed a decrease in sensitisation to Arah2 with an increase to Arah9 and Prup3 correlated with age.

## Conclusions

Peanut allergy is frequent in subjects with allergy of vegetal origin. Arah2 and Arah9 were the most relevant proteins. Arah2 predominated over Arah9 in younger patients. The reverse was observed in older patients. A strong association was found between IgE response to Arah9 and to Prup3.

